# The Book World of Medicine and Science

**Published:** 1911-06-24

**Authors:** 


### THE BOOK WORLD OF MEDICINE AND SCIENCE.

The Evolution and Function of Living Purposive Matter. By N. C. Macnamara, F.R.C.S. Vol. 97 of the "International Scientific Series." Edited by F. Legge. (London: Kegan Paul, Trench, Trübner and Co., Ltd. 1910. Pp. 298 + xi, with illustrations. Price 5s.)

Though issued at a price within the reach of even a moderately well-lined pocket, this volume by no means partakes of the qualities of a popular scientific treatise, for its author presupposes at least an elementary knowledge of biology and human anatomy in his readers. To one possessed of these qualifications the perusal of this work will be a pleasant task, for the author is at all times interesting and informative. The earlier chapters of the book are devoted to proving his contention that the purposive elements of protoplasm undergo evolution *pari passu* with those elements which constitute the structures and organs of the bodies of the ascending classes of animals. The development of these elements through various stages is traced, with the object of showing that this specialised form of matter has come to occupy a definite part of the cerebrum. After demonstrating that the psychic nervous substance of the brain has been developed from matter possessing instinctive functions, he argues that mental phenomena are brought into operation through the orderly working of such a system. He believes that in the majority of human beings inherited, instinctive, and emotional faculties exert a paramount influence over their personal characters, and supports the soundness of his conclusions by giving the second part of the book over to a description of the leading characteristics displayed by a long line of individuals who lived under conditions well adapted to show the power exercised by their leading characteristics on the actions of many succeeding generations, and on the destinies of the race of which they were members. For this purpose the origin and racial character of a tribe of Celts who about 450 A.D. settled in the West of Ireland are described at some length. Since their descendants remained on the same lands, in a fairly constant environment, and uninfluenced by admixture with other races until the end of the seventeenth century, this tribe is peculiarly adapted to serve as an illustration of the truth of the author's contentions. It is impossible in the short space at our disposal to follow him through his fascinating description of this people, and their fortunes through the succeeding centures. The inquisitive reader who takes the trouble to read the original will be well rewarded for his pains. We cannot refrain, however, from quoting a few pasages illustrative of the status of the medical practitioner in those early times. The author, writing of those who followed the Brehon Code long before the Christian Era, states that "the physician was much esteemed and . . . it was not uncommon for a tribe to make him a grant of land so that he was prevented from being disturbed by the cares and anxieties of life, and enabled to devote himself to his profession. It is stated that if an unlawful 'physician' removes a joint or sinew without obtaining an indemnity against liability to damage, and without notice that he was not a regular physician, he is subject to a penalty with compensation to the patient. If a man was maliciously wounded it was the duty of the physician to certify the same, and on his judgment compensation was awarded." The author goes on to remark that the value of cleanliness, pure water, and fresh air was well known to those physicians. Their houses, in which were treated all the sick and wounded from the chiefs downwards, were built either on the banks of a running stream or with a stream passing through the precincts. Each house had to be provided with four doors, so that all that took place within was open to inspection "and to permit one door being left open whichever way the wind blew." The hot-air bath was much employed for rheumatism, and shampooing is highly extolled. Thus once again it is shown that there is nothing new under the sun, and that the fresh-air "fad" existed in pre-Christian times!

A Manual of Nursing. By Margaret F. Donahoe. (New York and London: Appleton and Co. 1910. Pp. 490. Illustrated. Price 7s. 6d. net.)

In this new manual the nurse will find a very large amount of medical and surgical knowledge, as well as an excellent exposition of the principles of nursing proper. Miss Donahoe, as the matron of the Philadelphia General Hospital, has had experience in the training of nurses and the lectures of physicians and surgeons of the very best kind, and it is only to be expected that in a manual compiled by her one would find sound ethical teaching and technical knowledge of the most serviceable nature. We may be allowed to doubt, however, if this book possesses any material advantages for the English nurse over those excellent text-books already published in this country, while it has the disadvantage of discussing many details and methods which differ from those in common use in English hospitals, and of connecting strange names to familiar procedures. The subject-matter is generally excellent, and set out in a most readable manner; but we feel that this is hardly a manual to be recommended for probationers to start on, as the arrangement is not calculated to present the salient facts in an easily assimilable form. To the nurse who has already had a thorough grounding in anatomy and physiology this book should prove an exceedingly useful aid in revising her lectures and helping her to understand the rationale of her practical work. Indeed, we might well recommend the book to ward-clerks and dressers as a useful preliminary to enable them to take fuller advantage of their earlier opportunities. The scope of the manual is a wide one, and any blemishes to be found are chiefly in the more strictly medical portions, and are, perhaps, inseparable from a book in which it is difficult to allot adequate space to purely medical points, but the chapters on the nursing of different classes of diseases are deserving of the highest commendation.

Hints on Outfit for Travellers in Tropical Countries. By Charles Forbes Harford, M.A., M.D. (London: The Royal Geographical Society. 1911.)

This short work should prove of the greatest assistance to those contemplating a journey to any of the tropical countries. The author, who has had much experience of the difficulties attending the traveller in various tropical climes, gives valuable, if concise, information on clothing, camp equipment, canteen and cooking requisites, provisions, protection from mosquitoes and parasites, boxes and packing, stationery and writing materials, medicine and medical requisites, water purification, toilet requisites, guns, rifles, and ammunition, and indicates the way in which further knowledge on particular points may be acquired. It is interesting to note that under the authority of the Council of the Royal Geographical Society arrangements have been made for the instruction of intending travellers in any or all of the following subjects: Surveying and mapping, geology, botany, zoology, anthropometrical measurements, photography, meteorology, and outfit, at a cost of half-a-crown per lesson of one hour's duration.

A Text-Book of Gynæcological Surgery. By Comyns Berkeley, M.D., F.R.C.P., and Victor Bonney, M.D., F.R.C.S. With 392 drawings by V. Bonney and 16 coloured plates. (London: Cassell and Co., Ltd. 1911. Pp. 720. Price 25s. net.)

The present reviewer cannot recollect any book, either on gynæcology or on operative surgery, which has given him greater pleasure to read through than this new and important text-book. In every respect it reaches a standard so high that it stands without a rival in the special field to which the authors have chosen to limit their enterprise. Many admirable text-books of operative surgery contain special chapters or sections on pelvic surgery, written usually by those who specialise in this branch of work; but it is very seldom that the accounts of gynæcological surgery thus compiled are entirely satisfactory. For one thing, the contributor is limited as to space, illustrations, treatment of his subject, and in other ways by the editor who has asked him to contribute; for another, the greater part of the particular volume in which the contribution appears may deal with operations on regions which no gynæcologist wants to operate on, and thus the latter has to pay for an expensive book only part of which he wants to read. But apart from this there is no authority which deals so lucidly, so practically, and so thoroughly with the subject of operative gynæcology as does the work now under consideration.

In technique the authors describe chiefly those details of instruments, sutures, and methods generally which they themselves adopt; alternative usages they for the most part ignore. This is a wise plan, though it must inevitably result in the omission of procedures that individual readers have found helpful. Still a text-book which fails to be authoritative, even dogmatic, on such points often fails to carry due weight. In respect of the operations themselves this criticism cannot be made; every operation now in common usage is described, even when the authors themselves do not practise it. The indications for, merits of, and objections to, each operation are discussed along with the description of how it is performed. These discussions are excellent, full of sane and surgical common-sense. Indeed, throughout the volume it is always made perfectly plain, not only what the authors recommend, but why they recommend it; they are always ready to give ample reasons for their articles of surgical religion, and to back up their conclusions.

To take a few points of interest in detail. In the first chapter are some excellent remarks on speed in operating, a factor in success which too many dawdling surgeons still neglect to the detriment of their patients; at the same time it is pointed out that the speed maniac who will not trouble to stop all oozing before closing his wound is equally well failing to do his best for those in his charge. In the model suite of operating rooms described there is a good deal of waste space; the plans do not seem to be anywhere near ideal. Also the anæsthetist's room is unprovided with a wash-basin, and to transfer the anæsthetised patient from it to the theatre a corridor has to be crossed, which seems quite a bad principle. In dealing with the preliminary treatment of operation cases, the authors omit altogether the toilet of the mouth, a most important matter which rarely receives the attention it deserves, particularly in hospital practice where there is most need for it. They order strychnine injections hypodermically twice a day for four days prior to any major operation, a custom which some operators defend as enthusiastically as others denounce it. Among the surgeon's armament of preparation no mention is made of sterilised stockingette sleeves, which are becoming steadily popular. Sims' speculum the authors do not use, preferring Auvard's for every sort of case. Michel clips, which very many operators have now abandoned, are warmly praised. Several methods of suture are described and figured, but the subcuticular stitch is not mentioned at all, though various other procedures for minimising the unsightliness of scars are considered.

On curetting and the indications for it there is an excellent section, full of the sound sense for which the whole work is noteworthy. Hysterectomy is even better treated, perhaps because the subject is larger and many sided. There is an excellent *résumé* of the arguments for and against total and subtotal hysterectomy respectively. While following Mr. Bland Sutton's practice themselves, the authors recognise that there are still surgeons of wide experience who prefer the total operation. The extra difficulty of the latter is, in the present writer's opinion, very generally underestimated. Drs. Berkeley and Bonney set down the extra time required as five to fifteen minutes; from watching the operations of several London surgeons it would seem that twenty to forty minutes is nearer the mark. It is the small vessels in the cut edges of the vagina and the adjacent parametrium which cause this difficulty; oozing down at the bottom of the deep chasm is not easily stopped, and from statistics given by the authors in another chapter it would appear that the blood lost in the subtotal operation averages about nine ounces against eleven and a half for total hysterectomy. Schauta's operation is especially well described, though it is recommended only for certain exceptional cases. A very full and philosophical discussion of Wertheim's operation, and of everything connected with it, is given; every point is illustrated with the latest Continental statistics, which deal with hundreds, even thousands, of cases. The two most novel features are the Berkeley-Bonney special clamp for the vagina and the use of the cautery-knife for dividing this structure. The necessity of routine irrigation of the bladder to minimise risk of cystitis is insisted upon.

After-treatment and post-operative complications are thoroughly dealt with; in fact, a hundred and thirty pages are allotted to this very important subject. The authors keep their patients a good long time in bed after operations and have not adopted Continental theories and practice in this respect. It may be noted that they allow nothing by the mouth, even in minor operations, until six hours afterwards; this rule appears unnecessarily harsh for short operations.

Finally, the illustrations demand notice. The coloured plates of morbid pelvic conditions are faithful and commendable. But Dr. Bonney's line drawings require perhaps higher praise than this. They are of no great artistic merit, but they do illustrate in an almost perfect way the successive stages and manipulations of the operations whose descriptions they accompany. The text is wonderfully explicit; but with these wholly admirable drawings in addition the chance of misunderstanding is to all intents eliminated. The labour of making nearly four hundred of these illustrations must have been so enormous that it gives real pleasure to be able to testify to the splendid results achieved. Finally, it may be added that, with occasional exceptions, the text is scholarly. Probably this attention to composition is one of the secrets of the ease with which the book can be read and understood. The authors have in this volume done credit both to themselves and to British gynæcology. They are to be congratulated on a fine piece of work, in the preparation of which, it is also evident, the publishers have done their part extremely well.

